# 3‐Hydroxyisobutyryl‐CoA hydrolase deficiency in an Iranian child with novel HIBCH compound heterozygous mutations

**DOI:** 10.1002/ccr3.1998

**Published:** 2019-01-15

**Authors:** Parvaneh Karimzadeh, Mohammad Saberi, Kobra Sheidaee, Mitra Nourbakhsh, Mohammad Keramatipour

**Affiliations:** ^1^ Department of Pediatric Neurology Mofid Children Hospital Shahid Beheshti University of Medical Sciences Tehran Iran; ^2^ Department of Medical Genetics School of Medicine Tehran University of Medical Sciences Tehran Iran; ^3^ Department of Biochemistry Faculty of Medicine Iran University of Medical Sciences Tehran Iran

**Keywords:** HIBCH deficiency, isovaleric acidemia, Leigh‐like disease, mitochondrial disorders, valine metabolism

## Abstract

We report a patient presenting with developmental delay, Leigh‐like abnormalities on MRI and elevated 3‐hydroxyisovaleric acid levels. Upon whole‐exome sequencing, he was diagnosed with 3‐hydroxyisobutyryl‐CoA hydrolase (HIBCH) deficiency, and hence subjected to specific dietary treatment. HIBCH deficiency should be considered in the differential diagnosis of Leigh‐like disease and/or organic aciduria.

## 1 INTRODUCTION

3‐hydroxyisobutyryl‐CoA hydrolase (HIBCH) (a nuclear‐encoded mitochondrial enzyme) deficiency is one of the rare inborn metabolism errors in valine's catabolism with an estimated incidence of about 1:128 000 in Asia. Main clinical manifestations include childhood neurodevelopmental and motor delay, dystonia and ataxia, which are associated with brain imaging abnormalities. Due to few cases which are reported worldwide, the characteristics, genes, etc. are not well defined. Thus, it is important to pay attention to cases which are suspected of HIBCH deficiency.

We report a patient which has no positive family history of metabolism disorders or consanguineous marriage of parents which have developmental delay and Leigh‐like abnormalities showing on magnetic resonance imaging (MRI), as HIBCH deficiency with elevated 3‐hydroxyisovaleric acid in urine and HIBCH mutation in whole‐exome sequencing. Two novel heterozygous missense variants (c.641C>T; p.Thr214Ile and c.913A>G; p.Thr305Ala), which are detected in HIBCH gene (NM_014362.3), are responsible for hydrolysis of both 3‐hydroxyisobutyryl‐CoA (HIBYL‐CoA) and 3‐hydroxypropanoyl‐CoA, which are important in valine catabolism pathway. HIBCH deficiency, as a disorder of valine catabolism, should be considered in the differential diagnosis of Leigh‐like disease and/or organic aciduria. Due to the rare cases of this disorder, proper diagnosis, and attention to diagnostic clues are essential.

Mitochondrial disorders are rare generally, accounting for about 1:5000 births,^1^ half of which originate from mitochondrial or nuclear‐encoded defects of mitochondrial protein synthesis, which is caused by rare inborn metabolism errors^2^; 3‐hydroxyisobutyryl‐CoA hydrolase (HIBCH) is a nuclear‐encoded mitochondrial enzyme and its deficiency (OMIM #250620) impairs conversion of 3‐hydroxyisobutryl‐CoA to 3‐hydroxy‐isobutyric acid, as the fifth step in valine catabolism, accumulating toxic valine metabolites that interfere with mitochondrial enzymes potentially.^3^, ^4^

This autosomal recessive disease is a very rare condition with an estimated incidence of 1 in 551 545 in Europeans to 1 in 127 939 in South Asia.^5^ Since its introduction in 1982,^6^ few cases have been reported worldwide, the results indicated main clinical manifestations including neurodevelopmental and motor delay, dystonia and ataxia, in early infancy or within the first year of life.^5^, ^7^, ^8^ Magnetic resonance imaging (MRI) shows a wide range of abnormalities, including involvement of the basal ganglia and cerebral peduncles with varying degrees of white matter atrophy.^5^, ^7^ Nevertheless, the cerebral changes which are indicated on MRI or computed tomography (CT) scan cannot differentiate between mitochondrial encephalomyopathies which are caused by primary respiratory chain (RC) deficiencies; therefore, diagnosis is only established by genetic and molecular assessment.^7^

Yet, as few cases are reported in the literature so far, it is important to pay attention to a more common Neuro‐metabolic condition, which is called Leigh syndrome (OMIM #256000),^8^ which has similar clinical features (infantile onset of progressive encephalopathy) and imaging characteristics (bilateral basal ganglia and white matter changes), which are caused mainly by defects in oxidative phosphorylation and may be accompanied with elevated lactic acid.^9^, ^10^ Thus, most patients manifesting such symptoms, are anticipated as mitochondrial diseases, firstly are diagnosed as Leigh's disease, while HIBCH, as a much rarer condition, is not investigated typically.^10^ Accordingly, it is important to pay specific attention to HIBCH deficiency in patients manifesting Leigh‐like signs and symptoms.^5^, ^12^ Furthermore, most studies have reported cases in siblings or children of parents with consanguineous marriage^5^, ^14^; in this paper, we report a patient which has no positive family history of metabolism disorders or consanguineous marriage of parents, manifesting developmental delay and Leigh‐like abnormalities based on MRI, diagnosed as HIBCH deficiency with elevated 3‐hydroxyisovaleric acid in urine and HIBCH mutation in whole‐exome sequencing (WES).

## 2 CLINICAL REPORT

The patient was the first child of healthy parents, without consanguineous marriage, which was born in Qom, Iran. He was born at 38 weeks of gestation after a normal pregnancy. Growth parameters were normal at birth time (birth weight: 3000 g, birth height: 50 cm, and head circumference (HC): 34 cm). Neonatal screening revealed no pathologies which was performed using tandem mass spectrometry. He only had a history of hospitalization due to neonatal jaundice.

**Figure 1:**
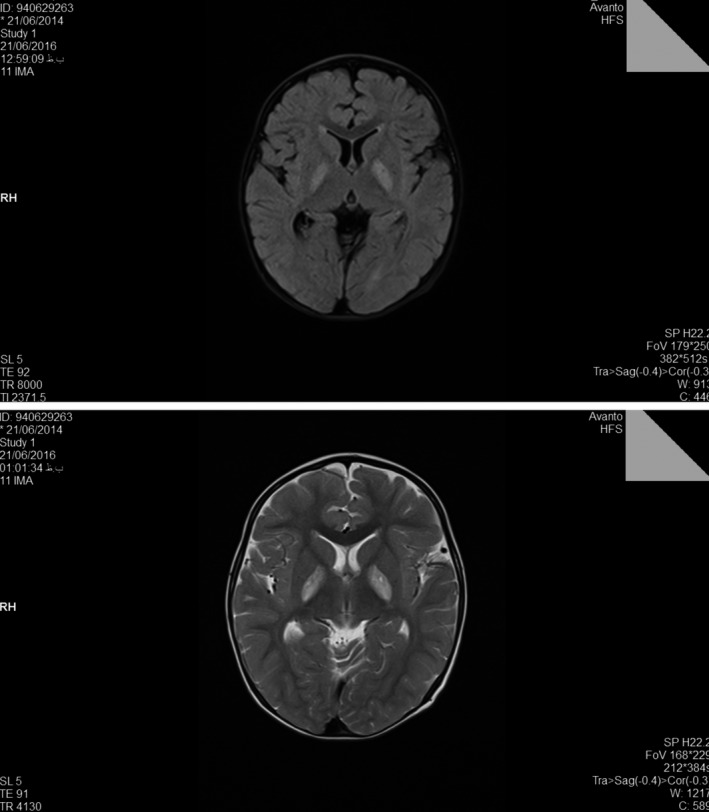
Brain MRI at age 2 y showed bilateral high signal lesions in the globus pallidus

At age of 15 months old, the child had recurrence attacks following febrile diseases with symptoms including weakness, myoclonus, and eye nystagmus. The results of brain MRI which was requested for the patient at age of 2 years old revealed symmetric signal abnormalities of the basal ganglia, reminiscent of metabolic or mitochondrial disorders (Leigh‐like syndrome) without any structural brain anomalies, myelination defect, or heterotopia (Figure 1).

At the age of 3.5 years old, the patient neither could walk independently, nor talk naturally (could not make a sentence). In physical examination, he exhibited hyperactive deep tendon reflexes with general muscular hypotonia, which is a central coordination disorder, ataxia, dysmetria, convergent strabismus and nystagmus of the eyes, and growth parameters were delayed (Length: 90 cm (3%), weight: 12 kg (3%), and HC: 48.5 cm (15%)).

Further laboratory examinations revealed that plasma amino acids and plasma acylcarnitine profile were normal. The organic acid in the urine showed an elevated 3‐hydroxyisovaleric acid (110 mmol/mol creatinine, normal <44 mmol/mol creatinine). Serum biotinidase activity was normal. Blood lactate and ammonia were normal in repeated measurements.

## 3 MOLECULAR GENETIC ANALYSIS

### 3.1 DNA extraction

Blood samples were collected from the patient and his parents in tubes containing ethylene diamine tetra acetic acid (EDTA). Genomic DNA was extracted from whole blood sample using Blood SV mini kit (GeneAll Biotechnology Co., LTD, Seoul, South Korea) according to the manufacturer's instructions. Concentration and purity of DNA were assessed by using a microspectrophotometer before being used for PCR‐Sanger sequencing.

Another round of quality control for DNA sample was performed before using DNA for WES. Quantity of DNA was measured by PicoGreen (Invitrogen, Thermo Fisher Scientific, Waltham, MA, USA) using Victor3 fluorometry. DNA condition was assessed by gel electrophoresis.

### 3.2 Polymerase chain reaction (PCR) and Sanger sequencing

All targets which were amplified by PCR and PCR products were purified using PCR SV mini kit (GeneAll Biotechnology Co., LTD). Sanger sequencing of PCR products was performed using Applied Biosystems 3500 Genetic Analyzer (Pishgam Biotech Co., Tehran, Iran). The obtained sequences were analyzed by comparing them to the reference human genome sequence.

### 3.3 Whole‐exome sequencing

Library preparation was performed by using Agilent SureSelect Human All Exon V6 system (Agilent Technologies, Inc., Santa Clara, CA, USA) according to manufacturer's instructions. Libraries were sequenced by high‐throughput paired‐end sequencing using HiSeq 4000 sequencing platform (Illumina Inc., San Diego, CA, USA). The sequencing was offered as a service by Macrogen Inc., Seoul, South Korea.

Sequencing short reads of 101‐base long were aligned to the reference human genome hg19 from UCSC genome browser (University of California, Santa Cruz, CA, USA) using the Burrows‐Wheeler Aligner (BWA) program. Variant calling and filtering were done using Genome Analysis Toolkit (GATK‐v3.4.0). Detected variants were annotated using SnpEff‐v4.1g software (Cingolani et al., 2012)^11^.

Then, proper filtering and interpreting a short list of variants in terms of pathogenicity were performed based on ACMG (American College of Medical Genetics and Genomics) guidelines for variant interpretation.

In order to evaluate the pathogenicity of novel variants, the potential effect of a given variant on the function or structure of the encoded protein was analyzed. The analysis was carried out based on conservation, physical properties of the amino acids or possible occurrence in regulatory or splicing motifs using bioinformatics tools. OMIM and PubMed were reviewed for previous publications which are related to the candidate causative gene.

## 4 RESULTS

Isovaleric Acidemia (IVA) was suggested as the possible diagnosis for the patient based on the analysis of urine organic acids that showed an elevated 3‐hydroxyisovaleric acid level and that serum biotinidase activity was normal. Therefore, PCR‐Sanger sequencing was used in order to analyze all exons and exon‐intron boundaries of IVD gene (NM_002225). No pathogenic variant was detected in this evaluation.

As no specific diagnosis was available, WES was used in order to evaluate all known exons and their flanking regions. Total number of reads which are obtained from this sample was equal to 141 209 836. These reads generated total sequence of more than 14.26 gigabases that gave an average throughput depth about 235.9 for target regions; 99.9% of target regions had a coverage of more than 1X, and 98.9% showed the coverage of more than 10X.

By using WES, two heterozygous missense variants (c.641C>T; p.Thr214Ile and c.913A>G; p.Thr305Ala) were detected in HIBCH gene (NM_014362.3). Multiple lines of in silico obtaining from computational analysis support the deleterious effect of the variants on the gene or gene product.

**Figure 2:**
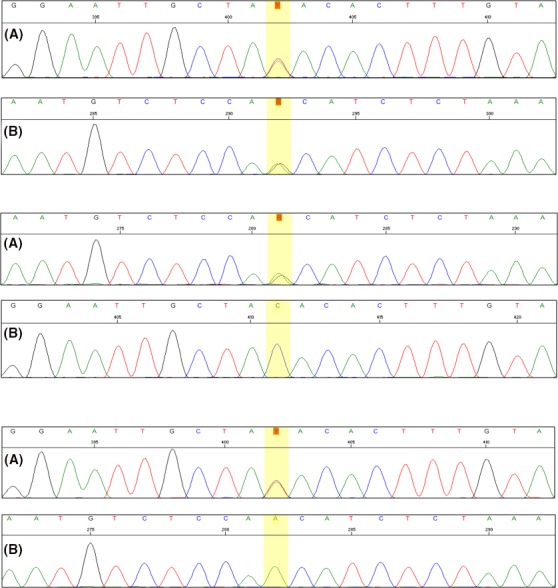
Sanger sequencing chromatograms of the HIBCH mutated regions in the patient (top) showing the heterozygous c.641C>T (A) and c.913A>G (B) variants, the patient's father (middle) showing the heterozygous c.641C>T variant (A) and the normal homozygous c.913A (B), and the patient's mother (bottom) showing the normal homozygous c.641C (A) and the heterozygous c.913A>G variant (B)

Compound heterozygosity of detected variants was confirmed by trio PCR‐Sanger sequencing in the patient (Figure 2) and each of the parents. Evaluating the parents confirmed the presence of heterozygosity in his father for the variant c.641C>T and the presence of heterozygosity in his mother for the variant c.913A>G (Figure 2). Therefore, paternal and maternal variant origins were confirmed, with Trans configuration in the patient consistent with the observed loss‐of‐function phenotype.

## 5 DISCUSSION

In the present study, we reported a 4 years old boy with developmental delay, including inappropriate walking and talking, and growth parameters (Percentile). Abnormalities showing in brain imaging at the age of two years old showed bilateral high signal lesions in the globus pallidus. Nevertheless, patient was not diagnosed appropriately until the age of approximately 3.5 years old, when the child was suspected for having Isovaleric Acidemia after observing elevated urinary level of 3‐hydroxyisovaleric acid and serum biotinidase activity that was normal. Afterward, for confirming diagnosis, he was referred for genetic counseling. As no pathogenic variant was detected in PCR‐Sanger sequencing, which was used to analyze all exons and exon‐intron boundaries of IVD gene (NM_002225), WES was performed in order to find out the cause of disease in the patient, and the results revealed that there are two heterozygous missense variants (c.641C>T; p.Thr214Ile and c.913A>G; p.Thr305Ala) in HIBCH gene (NM_014362.3), which are responsible for encoding proteins in hydrolysis of both 3‐hydroxyisobutyryl‐CoA (HIBYL‐CoA) and 3‐hydroxypropanoyl‐CoA, and are important in valine catabolism pathway. Homozygote or compound heterozygote mutations in HIBCH gene cause 3‐hydroxyisobutryl‐CoA hydrolase deficiency (OMIM #250620) that is inherited in autosomal recessive manner. Both variants were novel and there was no previous report for their pathogenicity. They were absent in population's databases such as ExAc, 1000G, and dbSNP. Both were also absent in Pishgam Database of Genomic Variants in Iranian Population (Pishgam Biotech Co., Iran). Therefore, paternal and maternal origins and Trans configuration of variants were confirmed in the patient. Based on the available evidence, we assumed these variants as the cause of disease in the patient.

Although manifestations of HIBCH deficiency overlap with other mitochondrial disorders, its management is different and treating with carnitine and dietary valine and protein restriction appears to be beneficial. We started the treatment after definite diagnosis and the parents were introduced to a genetic counselor for future pregnancy.

**Figure 3:**
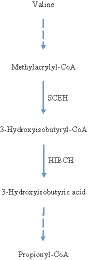
A summary of valine metabolism, highlighting the role of short‐chain enoyl‐CoA hydratase (SCEH) and 3‐hydroxyisobutyryl‐CoA hydrolase (HIBCH)

In metabolic pathway of valine, first, valine changes to methylacrylyl CoA in three stages; then, methylacrylyl CoA changes to 3‐hydroxyisobutyryl‐CoA by crotonase, and in the next step, 3‐hydroxyisobutyryl‐CoA changes to 3‐hydroxyisovaleric acid by HIBCH and after one other step, it turns to the end product propionyl CoA finally 4 (Figure 3).

HIBCH (EC 3.1.2.4) is a highly specific mitochondrial enzyme in monomeric form and in case of its deficiency, the toxic substrates accumulate in mitochondria that interfere with mitochondrial enzymes and deplete mitochondrial pools of cysteine, glutathione, thioredoxin, CoA, or lipoic acid.^3^, ^12^ The accumulated substrate of HIBCH can be detected in urine as 3‐hydroxyisovaleric acid conjugates,^13^, ^14^ like in conditions which there is an increased fatty acid oxidation with elevated activity of short‐chain enoyl‐CoA hydratase (SCEH) that lead to an increased production of 3‐hydroxyisovaleric acid.^7^ In our case, similarly, the analysis of urine organic acids showed an elevated urinary level of 3‐hydroxyisovaleric acid and IVA which was suggested as the possible diagnosis for the patient.

As far as we are concerned, <10 cases with HIBCH deficiency have been reported, all of which reported cases with consanguineous parents or positive family history, while our patient had none of these conditions. Reuter et al^10^ and Loupatty et al^15^ reported diagnosis of HIBCH deficiency in the first child of healthy consanguineous parents, and Ferdinandusse et al reported two brothers, which were born from Pakistani parents which were distant relatives.^16^ Nevertheless, our patient neither had family history of any metabolism disorder, nor was the child of a consanguineous marriage; although he was also the first child of the family, like the patients which were reported by Loupatty et al^15^ and Reuter et al,^10^ but contrary to them, our patient's parents did not have a consanguineous marriage.

Notably, studies have reported similar clinical symptoms, including different neurodegenerative disorders, nystagmus, and motor disorders, in addition to abnormalities in the basal ganglia showing in MRI, but as they include a wide range of symptoms with several differential diagnoses,^15^, ^17^ further laboratory examinations are requested to differentiate between Leigh‐like mitochondrial diseases. However, laboratory tests do not seem to have similar results between the cases which were reported; for example, some have reported normal blood lactate and ammonia levels,^15^ which is similar to our study, while others reported elevated blood lactate.^5^ According to Reuter et al, RC enzymes were at borderline in skeletal muscle and diagnosis was confirmed by WES (homozygous one‐base‐pair insertion in HIBCH)^10^; similarly in the present case, that WES was used for final diagnosis. According to Ferdinandusse et al RC enzymes were deficient in skeletal muscle and diagnosis was confirmed by using spectrophotometric enzyme assay (low HIBCH activity) in cultured skin's fibroblasts which were taken from both brothers and direct Sanger sequence analysis which demonstrated a novel homozygous missense mutation (c.950G>A; p.Gly317Glu) in the HIBCH gene.^16^ In the present study, WES revealed two novel heterozygous missense variants (c.641C>T; p.Thr214Ile and c.913A>G; p.Thr305Ala) in the patient.

Although all the cases which are described above are diagnosed as HIBCH deficiency in childhood or early infancy, there are few cases which are reported in adult and adolescents with mild symptoms.^17^

Very few cases of HIBCH deficiency have been reported worldwide and most cases, if diagnosed, they were misdiagnosed as other mitochondrial disorders like Leigh's syndrome. Thus, it is essential to report any case with HIBCH deficiency in order to add to the physicians’ knowledge about this disorder. In this paper, we reported a patient which was a rare case regarding two dimensions: first, the child neither had a family history of metabolism disorders nor consanguineous marriage of parents, second, he had two novel heterozygous missense variants (c.641C>T; p.Thr214Ile and c.913A>G; p.Thr305Ala), which were detected in HIBCH gene (NM_014362.3). Similar to other cases which are reported, clinical and brain imaging results were similar to previous reports, and HIBCH mutation was confirmed by WES. Due to the rare cases of this disorder, proper diagnosing and paying attention to diagnostic clues are essential.

## 6 CONCLUSIONS

HIBCH deficiency, as a disorder of valine catabolism, should be considered in the differential diagnosis of Leigh‐like disease and/or organic aciduria. Due to the rare cases of this disorder, proper diagnosing and paying attention to diagnostic clues are essential.

## AUTHOR CONTRIBUTION

PK: conceptualized the study and was responsible for patient care. MS: conceptualized the study and performed and interpreted genetic studies. KS: corresponding author, drafted the manuscript and was responsible for patient care. MN: interpreted biochemical studies. MK: performed and interpreted genetic studies.

## CONFLICT OF INTEREST

None declared.
